# Stereotactic Body Radiotherapy in Recurrent and Oligometastatic Head and Neck Tumours

**DOI:** 10.3390/jcm13113020

**Published:** 2024-05-21

**Authors:** Jodie L. M. Tham, Sweet Ping Ng, Richard Khor, Morikatsu Wada, Hui Gan, Alesha A. Thai, June Corry, Houda Bahig, Antti A. Mäkitie, Sandra Nuyts, Remco De Bree, Primož Strojan, Wai Tong Ng, Avraham Eisbruch, James C. H. Chow, Alfio Ferlito

**Affiliations:** 1 Department of Radiation Oncology, Olivia Newton-John Cancer and Wellness Centre, Austin Health, Melbourne 3084, Australia; 2 Department of Medical Oncology, Olivia Newton-John Cancer and Wellness Centre, Austin Health, Melbourne 3084, Australia; 3 GenesisCare, St Vincent’s Hospital, Melbourne 3065, Australia; 4 Department of Radiation Oncology, Centre Hospitalier de L’Université de Montréal, Montreal, QC H2X 3E4, Canada; 5 Department of Otorhinolaryngology-Head and Neck Surgery, Faculty of Medicine, Research Program in Systems Oncology, Helsinki University Hospital, University of Helsinki, 00100 Helsinki, Finland; 6 Department of Radiation Oncology, Leuven Cancer Institute, University Hospitals Leuven, 3000 Leuven, Belgium; 7 Department of Otolaryngology—Head and Neck Surgery, VU University Medical Centre, 1081 HV Amsterdam, The Netherlands; 8 Department of Radiation Oncology, Institute of Oncology, 1000 Ljubljana, Slovenia; 9 Department of Clinical Oncology, Li Ka Shing Faculty of Medicine, The University of Hong Kong, Hong Kong SAR, China; 10 Department of Radiation Oncology, University of Michigan Medicine, Ann Arbor, MI 48109, USA; 11 Department of Clinical Oncology, Queens Elizabeth Hospital, Hong Kong SAR, China; 12 International Head and Neck Scientific Group, 35100 Padua, Italy

**Keywords:** head and neck cancer, recurrent, reirradiation, oligometastatic, stereotactic body radiotherapy

## Abstract

The treatment of head and neck cancers (HNCs) encompasses a complex paradigm involving a combination of surgery, radiotherapy, and systemic treatment. Locoregional recurrence is a common cause of treatment failure, and few patients are suitable for salvage surgery. Reirradiation with conventional radiation techniques is challenging due to normal tissue tolerance limits and the risk of significant toxicities. Stereotactic body radiotherapy (SBRT) has emerged as a highly conformal modality that offers the potential for cure while limiting the dose to surrounding tissue. There is also growing research that shows that those with oligometastatic disease can benefit from curative intent local ablative therapies such as SBRT. This review will look at published evidence regarding the use of SBRT in locoregional recurrent and oligometastatic HNCs.

## 1. Introduction

Head and neck cancers (HNCs) are the seventh most common malignancy worldwide, accounting for 5% of new cancer diagnoses annually [1]. First-line treatment involves a combination of surgery and radiotherapy with or without systemic therapy. However, locoregional recurrence occurs in 15–50% of patients treated with curative intent and continues to be a significant cause of morbidity and mortality [2,3,4], with up to 80% of recurrences occurring in previously irradiated areas [5]. While salvage surgery remains the treatment of choice, factors such as tumour location and extent, patient comorbidities, and poor performance status leave only 20% of patients as operable candidates [6]. The prognosis is poor, with a median overall survival (OS) of 3 to 5 months without treatment [7]. The use of palliative chemotherapy alone can extend OS to 7 months, and with cetuximab, it increases to 10.7 months [8,9]. The addition of immunotherapy to chemotherapy has further raised the median OS to 13 months [10]. Reirradiation in these patients, with or without systemic therapy, offers an alternative means for long-term survival and even potential for cure. Doses of 60 Gy or more are necessary for salvage treatment and can yield similar results to salvage surgery [11]. However, in patients with recurrent HNCs who have already received high-dose radiation, reirradiation presents a real challenge due to the risk of exceeding normal tissue dose tolerances and consequent significant toxicity. There are studies reporting rates of 3.7% to 29% severe late toxicity, including carotid blowout, soft tissue and osteoradionecrosis, and oesophageal stricture [12,13]. Conventional reirradiation techniques, such as intensity-modulated radiotherapy (IMRT), have been shown to increase median OS by 8 to 15 months compared to those not treated with radiotherapy [14,15,16], but the proximity of nearby radiosensitive critical structures often restricts reirradiation dose. More recently, stereotactic body radiotherapy (SBRT) has emerged as an alternative technique in the reirradiation setting that allows for focused delivery of treatment while limiting dose and toxicity to surrounding normal tissue.

SBRT is a form of highly conformal radiotherapy that can deliver high ablative doses per fraction of typically 5 Gy or more, to well-defined, precise targets. With improved immobilisation devices, multiplatform imaging techniques, and image guidance, SBRT can deliver large doses of radiation over shorter treatment durations. The steep dose gradient created between the target and healthy tissue decreases the overall dose to organs at risk. Though SBRT’s molecular mechanism of action is not fully understood, it is theorised that these ablative doses of radiation have antiangiogenic effects through direct vascular endothelial damage; this in turn can overcome the hypoxic tumour microenvironments and radioresistant nature of recurrent cancer cells [17]. The activation of regional lymph node T cells prompting the eradication of tumour cells through CD8+ T-lymphocyte pathways is also believed to have a role [18]. SBRT has evolved to become a standard therapy in inoperable early-stage lung cancer [19] and is increasingly utilised in select prostate cancer cases.

For HNC treatment, SBRT has primarily been employed in the reirradiation setting. However, in the elderly and medically comorbid population, SBRT as a definitive treatment modality has also demonstrated benefits in increasing progression-free survival (PFS) and OS without significant toxicity [20,21]. Furthermore, changing paradigms in the management of metastatic cancer have led to growing interest in the role of SBRT within the subset of patients with oligometastatic disease.

In this review, we summarise the literature documenting SBRT in locoregional recurrent and oligometastatic HNCs and explore future challenges and opportunities for its use in this tumour population.

## 2. Materials and Methods

A literature search was performed using MEDLINE and PubMed databases for eligible English language studies from January 2000 to February 2023. The search was performed using the terms ‘head and neck cancer’, ‘recurrent head and neck cancer’, ‘head and neck cancer reirradiation’, ‘stereotactic body radiotherapy’, ‘stereotactic ablative body radiation’, and ‘oligometastatic head and neck cancer’. Additional relevant papers were identified through manual checking of references. Studies were included if they were full articles published in peer-reviewed journals; the primary radiation technique used was SBRT; patients were either reirradiation or oligometastatic HNC cases; patients had HNCs with predominantly squamous cell carcinoma histology; and OS, PFS, or LCR were reported. We excluded those reporting on primary untreated or polymetastatic HNC, or with large non-HNC oligometastatic populations. Articles were separated into two groups: The first group focused on SBRT in HNC patients, and the second on SBRT in oligometastatic HNC patients. Manuscripts discussing SBRT in recurrent HNCs were further subdivided according to whether concurrent systemic therapy was delivered, or if SBRT was compared with other modalities of treatment such as IMRT.

## 3. SBRT in Locoregional Recurrent HNC

### 3.1. SBRT Alone

An early study in 2006 by Voynov et al. involving 22 patients with recurrent HNCs used a median SBRT dose of 24 Gy (range 10–36 Gy) over five (range 1–8) fractions without systemic therapy [22]. The median survival was 12 months. The rate of local progression was rather high, with 2-year locoregional control (LRC) and OS of 26% and 22%, respectively. The relatively lower doses utilised may have contributed to these outcomes, in which they reported only one case of acute Grade 3 toxicity.

Iwata et al.’s paper looking at both hypofractionated and single-fraction SBRT in locally recurrent nasal and paranasal carcinomas reported 1-year LRC of 62% and OS 67% [23]. Notably, 50.1% of patients eventually developed subsequent local recurrences, and 37.2% developed distant metastases within the median follow-up time of 21 months. Grade 3 or more toxicity was also higher than expected, at 23%, and one patient who received a single fraction of 20 Gy developed brain necrosis. Another study consisting of a more heterogenous HNC cohort using both hypofractionated and single-fraction treatment schemes documented similar 1-year LRC at 60.6%, and 15.9% had Grade 3 or more toxicity [24]. A Cyberknife study by Roh et al. using 30 to 40 Gy over three to five fractions on consecutive days observed relatively promising 2-year LRC and OS of 61.0% and 30.9%, respectively [25]. However, 16.7% of patients experienced severe Grade 4 toxicity, and one fatality occurred due to soft tissue necrosis.

Palliative hypofractionated SBRT of 30 Gy over five fractions in patients unsuitable for salvage surgery was well tolerated with minimal acute or late side effects [26,27]. Even with a large median tumour volume of 58.7 cm^3^ (range 8.5–211.3), complete response was seen in 25% and partial response in 31% of patients [26]. Khan et al. published the results of an elderly patient population with a median age of 87 years using a higher median dose of 40 Gy [27]. They reported a similar complete response rate but a higher partial response of 67%. One-year OS and LC were 60% and 50%, respectively.

When considering the use of SBRT for locoregional HNCs, it is important to acknowledge the limitations based on available evidence (Table 1). The majority of studies on this treatment approach have relied on small retrospective studies, typically ranging from 1 to 2 years. As a result, late side effects, such as soft tissue/mucosal necrosis and carotid blowout, are inevitably underreported. It is expected that these late side effects would increase with longer follow-up, potentially undermining the long-term benefits of SBRT. Despite the limitations, SBRT has demonstrated impressive short-term benefits and presents a viable treatment option for select patients who are not suitable for salvage surgery. Doses of 30 to 40 Gy appear to be associated with higher rates of LRC and OS compared to <30 Gy. Yet, the risk of significant Grade 4 or more toxicity in up to 16.7% of patients emphasises the need for careful patient selection. Due to the wide heterogeneity of HNCs and largely retrospective data, it has been difficult to determine exactly which patients are most suitable for reirradiation with SBRT. Current National Comprehensive Cancer Network (NCCN) HNC guidelines [28] regarding reirradiation with SBRT remain ambiguous but state that “the best outcomes are seen in patients with smaller tumours and no skin involvement. Caution should be exercised in cases of circumferential carotid artery involvement”. Patients should ideally be of reasonable performance status (ECOG 0 to 1), and prior radiotherapy should have been given at least more than 6 months from the appearance of the new disease.

### 3.2. SBRT with Salvage Surgery Plus/Minus Systemic Therapy

Salvage surgery remains the preferred curative intent treatment for patients with resectable locoregional recurrences. The role of adjuvant reirradiation is less defined and has largely been informed by small prospective and retrospective studies using conventional radiotherapy techniques. A comprehensive review that looked at these series recommends that postoperative reirradiation should be considered in patients with high-risk histopathological features (close or positive surgical margins, extracapsular extension, etc.), with the understanding that there is a greater risk of treatment-related toxicity or death [29]. We present research findings specifically related to reirradiation with SBRT following salvage surgery, with or without systemic therapy in Table 2.

In patients who underwent salvage surgery before SBRT and/or received concurrent courses of systemic platinum-based chemotherapy or cetuximab, 2-year LRC rates ranged from 28% to 58% and OS from 16% to 62% [32,33,39,42]. With median doses ranging from 30 to 35 Gy, the use of 35 Gy or more was noted to yield better LRC [32,39], seen at 71% compared to 59% in patients who received less than 35 Gy in one study (*p* = 0.014) [32]. Treatment was generally well tolerated, with mostly Grade 3 or less toxicity. However, Unger et al.’s patient cohort, which had a median target volume of 75 cm^3^ (range 7 to 276), did report a 9% incidence of Grade 4 toxicity [33]. They acknowledged that their higher rates of severe reactions were likely due to larger tumour volumes. Overall patterns of failure in these studies report local failure occurring in 32% to 44% of cases, distant metastases in 24% to 44%, and 11% to 16% of patients developing both [32,33,39,42].

Two prospective Phase II studies showed SBRT with concurrent cetuximab to be a safe and effective salvage option for recurrent HNCs [38,43]. The reported outcomes included a median OS of 10 to 13.6 months, a 1-year OS of 40–58%, and a 2-year OS of 24%. Comet et al. described that the addition of cetuximab did not result in excess toxicity above Grade 3 [38]. They attributed their encouraging results to a homogenous SBRT dosing of 36 Gy in six fractions and limited tumour sizes (median 29 mm). A retrospective paper published the results of 60 patients using a similar SBRT dose schedule to larger target volumes [40]; median OS was comparable at 11.8 months, and 1-year OS was 47.5%. Grade 3 or more adverse effects were largely cutaneous reactions, occurring in 30% of patients. One death occurred due to a fatal haemorrhage and malnutrition.

A prospective Phase I trial by Heron et al. illustrated that dose escalation up to 44 Gy was well tolerated [30]. In studies that prescribed median doses of 40 to 45 Gy, variable 2-year OS was observed, at 20% to 80% [47,48,49]. Pellizzon et al.’s small study exploring adjuvant SBRT after salvage surgery in 11 patients displayed especially promising results, with an OS of 53.3% at 4 years [49]. Less than 24 months between reirradiation and salvage targets within the oral cavity were associated with worse outcomes. One of the largest retrospective studies observed that regional and distant control outside the reirradiation field significantly improved with the use of concurrent systemic therapy, with 88% versus 50% recorded in patients who received SBRT alone [48]. Interestingly, conventional chemotherapy was associated with the highest rates of regional control compared to immunotherapy or cetuximab. Tumour volumes of more than 20 cm^3^ conferred a poorer prognosis. While a toxicity-focused paper by Quan et al. indicated that SBRT within the range of 40 to 44 Gy in patients with a prior median reirradiation dose of 70 Gy appeared to be safe with no adverse reactions above Grade 3 [45], Diao et al. reported such toxicity in 15% of cases [48]. These occurred more frequently in those with mucosal recurrence or undergoing concurrent systemic therapy. Notably, six patients who developed lingual artery bleeds had reirradiation to the oropharynx. One patient died from bone and soft tissue necrosis complicated by abscess formation.

Rwigema et al. demonstrated that further dose escalation up to 50 Gy was feasible and associated with significantly better disease control [36]. LCR at 1, 2, and 3 years with 40 to 50 Gy was superior to 15 to 36 Gy (69.4% versus 51.9%, 57.8% versus 31.7%, 41.1% versus 15.9%, respectively). Even at these higher doses, no Grade 4 or above toxicities were recorded. The writers attribute their outcomes to dose administration every other day rather than consecutive days, which may have increased the tolerability of SBRT in the reirradiation setting.

In summary, studies examining reirradiation with SBRT after salvage surgery reveal diverse outcomes influenced by variations in patient selection and treatment approaches. Higher doses of 35 Gy or more (up to 45–50 Gy) appear to be associated with improved locoregional control. However, larger volumes exceeding 20 cm^3^ are linked to a worse prognosis and potentially higher toxicity. The optimal dose schedule remains uncertain, underscoring the importance of balancing radiation dose with volume effect. Mesko et al. recommended that planning target volume (PTV) delineations be based on target sites [50]. They suggested PTV margins of 2 to 2.5 mm for neck and mucosal target volumes, and skull base tumours should ideally have margins as small as 1.5 to 2.0 mm. Both ExacTrac and cone-beam CT (CBCT) were used for positioning agreement, and while the former alone was typically sufficient for the accurate positioning of skull base targets, the addition of daily CBCT enhanced precision for neck and mucosal targets, which tend to have higher rates of positioning errors.

Furthermore, there remains an absence of consensus recommendations related to acceptable dose summation and organ-at-risk (OAR) dose tolerance for reirradiation with SBRT in HNCs. Gogineni et al. defined dose constraints for SBRT reirradiation in OARs approaching their maximum tolerance following previous radiotherapy [51,52]. Maximum cumulative EQD2 values (using an α/β ratio of 3 Gy) were calculated based on primary treatment with 65 Gy and 30 fractions and SBRT reirradiation of 35 to 40 Gy in 5 fractions. These yielded EQD2 maximum dose constraints of <129 Gy for the carotid artery, <86.7 Gy for the optic chiasm, and <54 Gy for the spinal/cord brainstem. Following these constraints, their cohort of 60 patients demonstrated low rates of toxicity.

### 3.3. Rates of Severe Complications

Reirradiation in the head and neck region is associated with a potential for significant toxicity due to the presence of multiple vital structures. Of particular concern is carotid blow-out syndrome (CBOS), which can be a fatal complication, especially in a reirradiation setting. Overall, the reported rates of Grade 3 or higher acute and late effects ranged from 0 to 36% [22,25,26,30,32,34,36,37,39,42,44] and 0 to 24% [30,31,33,34,35,36,37,39,42,43,44,47], respectively.

A retrospective 2011 study of 34 patients showed that SBRT yielded a promising response rate of 61.9% and a 2-year OS of 50% in patients with and without prior irradiation [35]. Severe late complications (bleeding, ulceration, severe mucositis, and skin necrosis) arose in 18%, all of whom received prior radiotherapy, with ultimately two patients dying of carotid haemorrhage.

Cengiz et al. reported a high rate of treatment-related CBOS in 17.8% of their patients [34]. They acknowledged that their study included a number of cases with large tumour volumes invading the carotid artery, and consequently, this complication occurred only in patients wherein the carotid arteries received 100% of the prescribed dose. Similarly, Yamazaki et al. experienced a higher rate of CBOS than anticipated in 10.3% of patients, resulting in nine fatalities [46]. Those with ulcerated lesions were noted to have worse outcomes, and this was likely a predictor for poorer locoregional control. They determined that hypofractionated SBRT may be inappropriate in patients with large, ulcerated tumours as well as carotid invasion of more than 180 degrees. These studies emphasise the need for careful patient assessment in the reirradiation setting.

Yazici et al. demonstrated that dosing protocols with consecutive-day treatments were associated with higher rates of CBOS compared to every-other-day treatment frequency [41]. The median dose received by the carotid artery in patients with CBOS was 36.5 Gy (range 34–42.8), but CBOS did not occur in any patient with a maximum carotid artery dose of less than 34 Gy. One- and two-year OS was seen to be superior with every-other-day treatment compared to consecutive-day prescriptions (84% versus 42%, and 38 versus 23%, respectively). Furthermore, LC was also greater at 90.6% compared to 67.5%.

A 10-year single-institution study of 291 patients with a focus on severe toxicities was published by Ling et al. [44]. Acute Grade 3 or more toxicity occurred in 11.3% of patients. Notably, one patient with acute Grade 4 pharyngeal oedema required tracheostomy and gastrostomy tube placement. Another patient with acute lingual artery bleeding received successful embolisation. Acute Grade 4 dysphagia and laryngeal oedema occurred in one patient, who proceeded to have a total laryngectomy; their postoperative course was complicated by a mucous plug and subsequent cardiac arrest. Late Grade 3 or more toxicity was reported in 55 (18.9%) patients. Grade 5 late toxicities included CBOS in three (1.3%) patients, severe dysphagia in two (0.9%), and single cases of fatal laryngeal oedema (0.4%) and mucosal haemorrhage (0.4%). Those with nodal recurrences were noted to have a lower incidence of severe acute toxicities, whereas recurrences in the larynx and hypopharynx were found to have a significant association with more severe late toxicities compared to other HNC sites. In fact, all patients with laryngeal and hypopharyngeal recurrences experienced severe late toxicities.

Multiple SBRT reirradiation studies have shown nasopharyngeal HNCs to be a favourable histology associated with improved LRC and PFS [33,46]. However, nasopharyngeal-specific retrospective studies show particularly high rates of late Grade 4 and 5 toxicities. This is likely due to the proximity of tumour volumes to critical structures such as the brainstem, cranial nerves, and optic pathways. Wu et al. examined outcomes in both persistent and recurrent cases using median doses of 18 Gy and 48 Gy, respectively [53]. Severe late complications occurred in 17 (19%) of patients: 6 (7%) with nasopharyngeal mucosal necrosis and 3 (3%) with brain stem necrosis. Patients with persistent disease rather than recurrence, as well as smaller tumour volumes of 5 cc or less, had a better prognosis. Seo et al. specifically looked at 35 previously irradiated nasopharyngeal patients [31]. While 5-year OS was favourable at 60%, late Grade 4 and 5 toxicities were reported in five (14%) patients. Two (6%) patients experienced severe mucosal necrosis, and three (8%) cases had life-threatening haemorrhage; consequently, there were two (6%) treatment-related deaths.

Despite the limited evidence in this space, a review by Grimm et al. describes a summary of recommendations for minimising the risk of CBOS and other bleeding events in HNC SBRT reirradiation [54]: (1) delivery of treatments on non-consecutive days; (2) for five-fraction SBRT, in the reirradiated major vessel, the volume of D_0_._5cc_ should be restricted to less than 20 Gy; and (3) as patients requiring circumferential major vessel irradiation or with pre-existing skin necrosis are at especially high risk for complications, other radiation approaches (i.e., conventional or hyperfractionation) should also be considered. While these findings are not validated and currently undergoing ongoing evaluation, in five-fraction SBRT, these constraints can be used as a guide: carotid D_max_ should be to ≤42 Gy and D_50%_ to ≤32 Gy [55].

### 3.4. SBRT versus IMRT

As detailed in Table 3, we identified two retrospective studies comparing SBRT and IMRT in the HNC reirradiation setting. Vargo et al. described definitive reirradiation with SBRT or IMRT in recurrent and second primary HNCs [56]. When adjusted for factors such as intertreatment interval and feeding tube or tracheostomy dependence, there were no significant differences in OS or locoregional failure. Nevertheless, the IMRT group exhibited a higher incidence of acute Grade 4 toxicities, including fistula development, ICU admission, and life-threatening bleeding, compared to the SBRT group (5.1% versus 0.5%, *p* < 0.01). These findings suggest that while IMRT enables the treatment of larger tumour volumes and targets those at a higher risk of microscopic extension, the larger irradiation volume inevitably leads to increased toxicity. SBRT was seen to be beneficial in smaller tumour volumes of 25 cm^3^ or less, and for RPA class III patients with poor prognosis (organ dysfunction defined as the need for using a pretreatment feeding tube or tracheostomy), and it also potentially facilitated the addition of systemic therapies. Orlandi et al. showed that IMRT was superior to SBRT in class II patients (defined as those with unresected disease with disease-free interval (DFI) >2 years or DFI <2 years and without organ dysfunction) [57]: Two- and five-year OS were 83.5% vs. 64.1% and 64.3% vs. 23.3% in IMRT compared to SBRT, respectively. However, we note that no patients received SBRT doses of more than 30 Gy, which may account for the lack of clinical benefit seen in this study. These findings underscore the need for a tailored radiotherapy approach based on specific patient characteristics and tumour factors in the reirradiation setting.

## 4. SBRT in Oligometastatic Disease

Approximately 4 to 24% of HNCs are metastatic at the time of diagnosis [58], with distant relapses occurring in 5% to 47% of patients after primary treatment [59]. Hypopharyngeal and laryngeal cancers appear to show the highest frequency of metastatic recurrences [60]. Advances in positron emission tomography (PET), computed tomography (CT), and magnetic resonance imaging (MRI) have continued to improve the diagnosis of metastatic disease in HNCs. Common sites of distant metastases are lung (70–85%), bone (15–30%), and liver (10–30%), with a relatively low incidence of intracranial metastases (0.4–8%). Systemic platinum-based chemotherapy with immunotherapy or targeted therapy is the current standard of care in patients with metastatic HNCs [61]. However, in HNCs, several types of metastatic disease can be distinguished as associated with different rates of survival: oligometastasis (maximum of three metastatic foci in ≤2 anatomic sites; 35%), explosive metastasis (≥4 metastatic foci at one anatomic site; 20%), and explosive-disseminating metastasis (spread to ≥3 anatomic sites or >3 metastatic foci in two anatomic sites; 45%). In addition, (oligo)distant metastases can be synchronous and metachronous. Oligometastases have a better prognosis than a polymetastatic pattern, while metachronous distant metastasis occurrence with recurrence of the primary index tumour is associated with the most unfavourable prognosis [62]. There has been increasing use of ablative interventions in those with oligometastatic and oligoprogressive disease. These patients with a limited burden of distant disease have the potential for local control (LC) and even cure with metastasis-directed therapies such as surgery, radiofrequency ablation, and radiotherapy. Multiple Phase II studies in a range of tumour types support the impact of these ablative treatments on PFS and OS [63,64,65,66]; a delay in time to systemic therapy has also been observed [67]. While surgical metastectomy has historically been the modality of choice, SBRT is emerging as an alternate and safe option, particularly in medically inoperable patients. Unfortunately, data on metastatic HNC patients undergoing SBRT are rare and often pooled with other tumour types [68]. Although most authors consider a maximum of three metastases in one or two organs as oligometastases, different definitions of an oligometastatic state hamper the comparison of studies.

SABR-COMET was the first randomised Phase II trial of its kind, comparing SBRT to standard-of-care palliative treatment [64]. The oligometastatic state was defined as a maximum of five metachronous lesions with not more than three of them per organ, although most patients in the study (94%) had 1–3 metastatic deposits. Staging with FDG-PET was not mandatory, so the number of metastases may have been underestimated in some patients. Within a population of 99 patients with variable primary tumour types, it demonstrated a significant improvement in median survival from 28 to 41 months in the SBRT arm in patients with one to five metastatic lesions [64]. An extended follow-up study reported encouraging 5-year OS of 42.3% with SBRT versus 17.7% in the standard-of-care group [69]. The benefit observed in SABR-COMET came at the cost of increased Grade 2 or higher treatment-related toxicity (29% versus 9%), including Grade 5 adverse events (5% versus 0%), albeit with no impact on quality of life as measured using the FACT-G scores [70]. In a large cohort from a population-based, provincial cancer program in British Columbia, rates of Grade 2 or higher toxic effects were even lower (18.6%) [71]. SBRT was cost-effective for patients with one to five oligometastatic lesions compared with standard of care [72]. To find a threshold number of metastases beyond which ablative treatment offers no additional benefit, the SABR-COMET-10 trial was designed [73].

Unfortunately, HNC is under-presented in these studies and accounts for less than 10% of included cases. Metastasis-directed management does, however, have demonstrable benefits in select HNC cases [74]. A critical review of oligometastatic HNC patients reported 5-year survival rates exceeding 20% after pulmonary and/or hepatic metastectomy and 2-year survival of 35% with SBRT [75]. There also appears to be no significant difference in survival outcomes between surgical excision and ablative radiotherapy [76]. Table 4. Illustrates a summary of literature looking at SBRT in oligometastatic HNC. These results largely originate from retrospective studies, and there remains a paucity of prospective data.

Franzese et al. looked at 48 patients with oligometastatic HNCs and reported a 2-year LC of 70.2%, PFS of 20.0%, and OS of 67.1% [81]. Increasing age, worse performance status, and non-salivary primary and non-lung metastases were associated with reduced OS. Other studies reported a 2-year LC of 57% to 93.3% and OS at 43% [77,84]. The median time to progression (TTP) in the study by Bates et al. was particularly rapid at 0.5 years and was thought to be driven largely by distant progression; DFS at 2 years was only 14% [77]. Singh et al. documented promising LC results of 77.4% at 3 years [84]. Patients with favourable Karnofsky Performance Status (KPS), smaller lesions, and non-spinal disease had better survival outcomes. There were no Grade 3 or more toxicities.

In the setting of pulmonary-only oligometastatic HNC disease, Bonomo et al.’s experience of 27 patients with limited pulmonary metastases of SCC-histological type demonstrated a 2-year PFS of 21.6% [79]. The median TTP of 10 months, with 1- and 2-year rates of 56.2% and 35%, respectively, suggest that SBRT also has the potential to defer the need for systemic treatment. Another similar retrospective study showed an excellent 2-year LC of 94.4% and OS at 61.6% [80]. Notably, 68% of patients had SCC histology, while non-SCC consisted of largely papillary thyroid or adenoid cystic type. This could account for the comparatively high OS of this cohort, due to the more indolent course of these subtypes even in metastatic disease. SBRT was well tolerated in both studies, with no toxicities more than Grade 2 [79,80].

There have been few studies that have compared SBRT with other avenues of treatment in the metastatic HNC population. A comparison of SBRT and palliative radiotherapy in oligometastatic salivary gland HNCs illustrated superior LC at 1 year with SBRT (57.5 vs. 37.8%) [83]. A retrospective paper by Schulz et al. demonstrated that metastasis-directed treatment with either surgery and/or SBRT had a median survival time of 24 months, which was more than three times higher than untreated patients with potentially treatable distant metastases [78]. OS at 2 and 5 years was 21.7% and 3.5%, respectively. Primary tumour of the oral cavity as well as liver, bone, and mediastinal metastases were significant negative prognostic markers and associated with a higher risk of death.

McBride et al. conducted a prospective Phase II trial of metastatic HNCs to determine if the addition of SBRT to nivolumab resulted in a significant abscopal effect and consequent decrease in the size of non-radiated lesions following SBRT of other oligometastases [82]. Patients were randomised to groups treated with nivolumab alone or with nivolumab with SBRT prescribed at 27 Gy in three fractions. No significant abscopal effect was observed, nor was there any difference in OS or toxicities between the two groups. This may reflect a need for further patient characterisation to select those who may benefit from SBRT with concurrent immunotherapy treatment. Notably, the dose prescribed in this trial was slightly lower than those reported in other studies mentioned above.

The wide variety of results observed is consequent to the very heterogenous HNC population involving diverse primary and metastatic tumour sites, as well as histopathological subtypes. The SBRT doses delivered also vary depending on tumour site and institution practices. While this makes the interpretation of the data difficult, it appears that patients with favourable prognostic factors such as locoregionally controlled disease, limited numbers of metastases, pulmonary metastases, or virally associated (HPV and EBV) HNCs may benefit most from ablative treatments like SBRT [85]. Additional clinical trials will be instrumental in exploring optimal radiation dose, fractionation, and timing, especially in relation to systemic therapy.

## 5. Recommendations

Through a comprehensive review of the available literature, we have identified key recommendations to assist clinicians in determining the appropriateness of SBRT and its safe implementation for carefully selected head and neck cancer patients requiring reirradiation. It is important to note that these recommendations are based on the available literature, which mainly consists of retrospective studies with limited follow-up periods. Larger prospective studies are needed to establish more definitive guidelines for the optimal use of SBRT in HNC reirradiation.

### 5.1. Patient Selection

Ideal candidates for stereotactic body radiotherapy (SBRT) reirradiation should have good performance status with an ECOG score of 0–1 and minimal significant comorbidities or organ dysfunction. Patients should preferably have a gap of more than two years from their initial radiotherapy, but definitely more than six months from their previous treatment, to qualify as candidates for SBRT reirradiation. Tumour volume plays a significant role in determining suitability; smaller tumours of less than 20 to 25 cm^3^ are most appropriate for SBRT, as larger volumes are associated with higher toxicity rates and may be better suited for IMRT. Special caution should be taken for patients with circumferential carotid artery involvement or large, ulcerated tumours, as these conditions are associated with poor outcomes and increased risk of severe complications such as CBOS. Incorporating systemic therapy with SBRT, such as platinum-based chemotherapy or cetuximab, can significantly improve locoregional control. However, this should be carefully balanced against the risk of increased toxicity.

### 5.2. Dose Prescription and Target Volumes

Higher SBRT doses of more than 35 Gy and escalation up to 45 to 50 Gy are recommended for improved locoregional control compared to lower doses of 30 Gy. Fractionation schedules prescribed as every-other-day treatments are better tolerated and have lower rates of carotid blowout syndrome compared to consecutive-day treatments. It is recommended to target only the gross disease, omitting elective nodal irradiation during reirradiation. To achieve the high conformality crucial for SBRT while limiting normal tissue exposure, the prescription dose should typically be set to encompass 95% of the PTV, permitting hotspots exceeding 120% within the GTV. The goal should be for at least 100% of the target to receive 95% of the prescribed dose, with normalisation to either the 95% or 90% isodose surface depending on the proximity of organs at risk to the target volume. In terms of PTV delineation, we suggest that 1.5 to 2.0 mm may suffice for skull base tumours, while margins of 2.0 to 2.5 mm are suggested for neck and mucosal target volumes when daily CBCT guidance and positioning verification are utilised.

### 5.3. OAR Dose Constraints and Dose Summation

Maintaining strict dose constraints for OARs is crucial to prevent severe complications. For SBRT of 35 to 40 Gy delivered over five fractions in the reirradiation setting, we advise the following constraints (based on α/β ratio of 3 Gy): (1) maximum dose constraints for the carotid of <32.5 Gy (EQD2 129 Gy), optic chiasm <25 Gy (EQD2 87 Gy), and lens <5 Gy (EQD2 11 Gy); (2) the cumulative maximum EQD2 dose for spinal cord/brainstem should be limited to 54 Gy; (3) for the cochlea and larynx, a mean dose limit of 15 Gy should be applied. Careful treatment planning and consideration of OAR constraints are crucial to minimising severe toxicities in the reirradiation setting.

## 6. Future Directions

As imaging advances in CT, PET, and MRI will undoubtedly improve the detection of recurrent and distant disease, there is an increasing need to develop treatment paradigms to appropriately manage these patients. Most notably, the advent of immunotherapy has stimulated much interest in its potential synergistic effects when combined with radiotherapy. Preclinical data suggest that radiation can promote antitumour cellular immunity through mechanisms such as the activation of cytotoxic T cells [86], promoting the development and maturation of B-cells [87], and the induction of immunogenic cell death to induce adaptive immune responses [88]. An HNC lab study demonstrated that the concurrent administration of both 10 Gy of radiation and anti-PD-L1 antibody together resulted in more effective tumour killing than delivering either treatment alone [89]. Conversely, radiation may also create locally immunosuppressive microenvironments due to the clearance of radiosensitive lymphocytes [90]. Elective nodal irradiation in a mouse model was in fact seen to decrease the immune response systemically and dampened the effectiveness of combined radiation and immunotherapy [91]. It is clear that further research is required to better understand the relationship between these two treatment modalities; there are a number of prospective studies currently underway in this sphere. KEYSTROKE is a Phase II trial looking at SBRT with and without pembrolizumab in locoregionally recurrent or second primary HNCs [ClinicalTrials.gov Identifier: NCT03546582]. Another Phase II study, IMPORTANCE/Keynote-717, will be looking at pembrolizumab with or without SBRT in recurrent and/or metastatic HNCs after progression to platinum-based therapy [ClinicalTrials.gov Identifier: NCT03386357]. An observational study assessing proton-based SBRT with nivolumab in recurrent locoregional or metastatic HNC is ongoing [ClinicalTrials.gov Identifier: NCT03539198]. Other upcoming Phase I and II research includes treatment with both durvalumab and tremelumumab alongside SBRT to sites of limited disease. Preclinical studies are also suggesting that a novel dosing scheme, with personalised ultrafractionated stereotactic adaptive radiotherapy (PULSAR) delivering large ablative dose ‘pulses’ over weeks or months, may enhance the effects of single-agent immune checkpoint blockade [92].

Increasing interest in the applications of artificial intelligence (AI) holds tremendous potential to revolutionise SBRT planning and treatment workflows [93,94]. With the ability to process vast amounts of patient data and optimise treatment plans based on individual anatomical and tumour characteristics, AI-driven algorithms offer the promise of personalised and precise radiation therapy delivery. Moreover, AI-based predictive models can assist in forecasting treatment outcomes and potential toxicities, enabling clinicians to tailor treatment strategies to each patient’s unique needs and risk profile. As AI continues to evolve and integrate into clinical practice, it is poised to streamline SBRT planning systems, enhance treatment efficacy, and ultimately improve patient outcomes in HNC management.

## 7. Conclusions

HNCs encompass a therapeutically challenging group for clinicians due to the heterogeneity of patients and histopathological type. SBRT has emerged as an effective and well-tolerated treatment option in locoregionally recurrent and oligometastatic HNC cases, especially in those with smaller tumour volumes who are unsuitable for salvage surgery. However, careful patient selection through multidisciplinary discussion is required to determine suitability for this modality. Given that the majority of evidence has been limited to retrospective studies, further prospective and randomised data are needed to determine optimal SBRT timing, dose, and fractionation in reirradiation and limited disease settings.

## Figures and Tables

**Table 1:** Summary of studies looking at stereotactic body radiotherapy alone in the reirradiation of head and neck cancers.

Study	Nature of Study	No. of Patients	Median SBRT Dose, Gy (Range)	Median No. of Fractions (Range)	Median Target Volume, cm^3^ (Range)	Follow-Up, Months (Range)	Outcomes	Late Toxicity (Grade 3 or Higher), %
SBRT Alone								
Voynov et al. 2006 [22]	Retrospective	22	24 (10–36)	5 (1–8)	19.1 (2.5–140.3)	19 (11–40)	2-y LRC: 26% 2-y OS: 22%	Acute Grade 3: 4.5
Roh et al. 2009 [25]	Retrospective	36	30 (18–40)	3–5	22.6 (0.2–114.9)	17.3 (0.5–30)	1-y LRC: 61.0% 2-y LRC: 52.2% 1-y OS: 51.1% 2-y OS: 30.9%	Acute Grade 3: 36.1 Late Grade 4: 5.5 Late Grade 5: 2.7
Siddiqui et al. 2009 [24]	Retrospective	44	14–48	1–8	15.5 cm^3^ (1.7–155)	204 (44–1435)	1-y LRC: 60.6% 2-y OS: 14.3%	Grade 3: 6.8 Grade 4: 9.1
Iwata et al. 2012 [23]	Prospective	51	35 (20–41.5)	1–5	33.8 (3.1–204.9)	21 (12–52)	1-y LRC: 62% 1-y OS: 67%	Grade 3+: 23
Bonomo et al. 2014 [26]	Retrospective	17	30 (25–35)	5	58.7 (8.5–211.3)	7.5 (2–17)	Median PFS: 7 mo	Acute Grade 3: 5.9
Khan et al. 2015 [27]	Retrospective	21	35–40	5–6	Maximal diameter 3.7 cm (1–10)	8	3-mo LRC: 87.5% 6-mo LRC: 62.5% 1-y LRC: 50% 3-mo OS: 90% 6-mo OS: 70% 1-yr OS: 60%	-

SBRT: stereotactic body radiotherapy; GTV: gross tumour volume; LRC: locoregional control; OS: overall survival; PFS: progression-free survival; LC: local control; CBOS: carotid blowout syndrome.

**Table 2:** Summary of studies looking at stereotactic body radiotherapy with salvage surgery and/or systemic therapy in the reirradiation of head and neck cancers.

Study	Nature of Study	No. of Patients	Median SBRT Dose, Gy (Range)	Median No. of Fractions (Range)	Median Target Volume, cm^3^ (Range)	Follow-Up, Months (Range)	Outcomes	Late Toxicity (Grade 3 or Higher), %
SBRT with Salvage Surgery and/or Systemic Therapy								
Heron et al. 2009 [30]	Prospective, Phase I	31	25–44	5	44.8 (4.2–216.6)	-	Median OS: 6 mo	None
Seo et al. 2009 [31]	Retrospective	35	33 (24–45)	3–5	7.9 (2.6–64)	25 (2–81)	5-y OS: 60%	Late grade 4: 8.6 Late grade 5: 5.7
Rwigema et al. 2010 [32]	Retrospective	85	35 (15–44)	5 (1–5)	25.1 (2.5–162)	6 (1.3–39)	1-y LRC: 51.2% 2-y LRC: 30.7% 1-y OS: 48.5% 2-y OS: 16.1%	Acute Grade 3: 4.7
Unger et al. 2010 [33]	Retrospective	65	30 (21–35)	5 (2–5)	75 (7–276)	16	2-y LRC: 30% 2-y OS: 41%	Late Grade 4: 11
Cengiz et al. 2011 [34]	Retrospective	46	30 (18–35)	5 (1–5)	45 cm^3^ (3–206)	-	1-y PFS: 41% 1-y OS: 47%	Acute Grade 3: 4.4 Late Grade 3: 2.2
Kodani et al. 2011 [35]	Retrospective	34	30 Gy (19.5–42)	5 (3–8)	22.0 (0.7–78)	16 (3–51)	1-y OS: 70.6% 2-y OS: 58.3%	Late severe: 17.6
Rwigema et al. 2011 [36]	Retrospective	96	35 Gy (15–50)/5 (1–5)	5 (1–5)	24.3 cm^3^ (2.5–162)	14 (2–39)	40–50 Gy 1-y LRC: 69.4% 2-y LRC: 57.8% 3-y LRC: 41.1% 15–36 Gy 1-y LRC: 51.9% 2-y LRC: 31.7% 3-y LRC: 15.9% Both groups: 1-y OS: 58.9% 2-y OS: 28.4%	Acute Grade 3: 5.2 Late Grade 3: 3.2
Vargo et al. 2011 [37]	Retrospective	34	40 (17–50)	1–5	25.9 (4.5–10.39)	12 (3–55)	1-y LRC: 59% 1-y OS: 58.9% 2-y OS: 28.4%	Acute Grade 3: 12 Late Grade 3: 6
Comet et al. 2012 [38]	Prospective, Phase II	40	36 Gy	6	64.1 (4.7–295.6)	25.6	1-y OS: 58% 2-y OS: 24%	Grade 3: 10.3
Karam et al. 2012 [39]	Retrospective	18	30 (21–40)	5 (2–7)	-	12 (0–88)	2-y LCR: 53% 2-y OS: 39%	Acute Grade 4: 11 Acute Grade 5: 5 Late Grade 4: 22
Lartigau et al. 2013 [40]	Retrospective	60	36	6	-	11.4	1-y OS: 47.5%	Grade 3: 30.0
Yazici et al. 2013 [41]	Retrospective	75 Group 1: consecutive Group 2: every other day	30 (15–35)	Group 1: 5 (3–5) Group 2: 5 (4–6)	Group 1: 54 (4–214) Group 2: 46 (5–166)	-	Group 1: 1-y OS: 42% 2-y OS: 23% Group 2: 1-y OS: 84% 2-y OS: 38%	CBOS: 14.7
Kress et al. 2015 [42]	Retrospective	85	30 (16–41)	5 (3–5)	103 (7.5–645)	17.3 (0.3–67.8)	1-y LRC: 57.8% 2-y LRC: 28% 1-y OS: 51.1% 2-y OS: 24%	Acute Grade 3: 2.4 Late Grade 3: 2.4
Vargo et al. 2015 [43]	Prospective, Phase II	50	40–44	5	36.5 (3.6–209.2)	18 (10–70)	1-y PFS: 33% 1-yr OS 40%	Acute Grade 3: 6 Late Grade 4: 6
Ling et al. 2016 [44]	Retrospective	291	44 (16–52.8)	5 (1–13)	29 (0.8–209.2)	-	1-y OS: 41.1% 3-y OS: 16.6% 5-y OS: 10.8% 10-y OS: 3.6%	Acute Grade 3+: 11.3 Late Grade 3+: 18.9
Quan et al. 2016 [45]	Retrospective	18	40 (40–44)	5	24.4 (4.4–75.7)	-	-	Grade 3: 5.5
Yamazaki et al. 2016 [46]	Retrospective	107	30 (15–39)	5 (3–8)	28.4 (1–339)	15 (10–122)	1-y OS: 55% 2-y OS: 35%	Grade 3+: 21
Stanisce et al. 2018. [47]	Retrospective	25	40 (24–44)	5 (3–5)	31.75 (5.5–121.8)	-	1-y OS: 32% 2-y OS: 16%	Acute Grade 3: 4 Late Grade 3: 6
Diao et al. 2021 [48]	Retrospective	137	45 (36–47.5)	5 (4–5)	16.9 (1.47–108.09)	-	1-y LC: 78% 1-y PFS: 47% 2-y PFS: 32% 1-y OS: 78% 2-y OS: 62%	Grade 3+: 15
Pellizzon et al. 2022 [49]	Retrospective	11	40 (30–48)	3 (2–6)	-	18 (5.2–71.1)	2-y OS: 80.0% 4-y OS: 53.3%	None

SBRT: stereotactic body radiotherapy; GTV: gross tumour volume; LRC: locoregional control; OS: overall survival; PFS: progression-free survival; LC: local control; CBOS: carotid blowout syndrome.

**Table 3:** Summary of studies looking at stereotactic body radiotherapy versus intensity-modulated radiotherapy in the reirradiation of head and neck cancers.

Study	Nature of Study	No. of Patients	Median Dose, Gy (Range)	Median No. of Fractions (Range)	Median Target Volume, cm^3^ (Range)	Follow-Up, Months (Range)	Outcomes	Late Toxicity (Grade 3 or Higher), %
SBRT vs. IMRT								
Vargo et al. 2018 [56]	Retrospective	IMRT: 64 SBRT: 64	IMRT: 60 (40–72) SBRT: 40 (16–50)	IMRT: 33 (12–60) SBRT: 5 (1–8)	IMRT: 30 (2–515) SBRT: 30 (1–427)	IMRT: 8.4 (0–130) SBRT: 7.1 (1–120)	IMRT 2-y OS: 35.4% SBRT 2-y OS: 16.3%	IMRT: Acute Grade 3: 16.6 Late Grade 3: 12.4 SBRT: Acute Grade 3: 11.7 Late Grade 3: 11.6
Orlandi et al. 2019 [57]	Retrospective	IMRT: 100 SBRT: 38	IMRT: 45–70 SBRT: 30	IMRT: 22–35 SBRT: 5	32.1 (15.6–69)	49.9 (28.9–86.3)	IMRT: 2-y OS: 83.5% 5-y OS: 64.3% SBRT: 2-y OS: 75.7% 5-y OS: 43.5%	IMRT + SBRT: Late Grade 3+: 17.6

SBRT: stereotactic body radiotherapy; mo: months; PFS: progression-free survival.

**Table 4:** Summary of stereotactic body radiotherapy studies in oligometastatic head and neck cancer.

Study	Nature of Study	No. of Patients	Median Age, Years (Range)	Sites Treated, %	Follow-Up, mo	Outcomes	Toxicity (G3 or More), %
Bates et al. 2018 [77]	Retrospective	27	65 (20–76)	Lung: 59.3 Spine: 22.2 Soft tissue: 18.5 Non-spine bone: 14.8 Liver: 3.7	19.2 (2.4–62.4)	1-y DFS: 27% 2-y DFS: 14% 1-y OS: 78% 2-y OS: 43%	-
Schulz et al. 2018 [78]	Retrospective	143				2-y OS: 21.7% 5-y OS; 3.5%	
Bonomo et al. 2019 [79]	Retrospective	27	67 (37–85)	Lung only	22 (6–73)	1-y DFS: 66.6% 2-y DFS: 21.6%	No Grade 3 or more
Pasalic et al. 2020 [80]	Retrospective	82	65 (26–93)	Lung only	20 (9–97.6)	1-y LC: 97.8% 2-y LC: 94.4% 1-y OS: 74.8% 2-y OS: 61.6%	No Grade 3 or more
Franzese et al. 2021 [81]	Retrospective	48	70 (32–83)	Lung: 59.1 Bone: 15.5 Lymph node: 14.1 Liver: 7.1 Adrenal: 4.2	20.2 (3–92.3)	1-y LC: 83.1% 2-y LC: 70.2% 1-y OS: 81% 2-y OS: 67.1%	No Grade 3 or more
McBride et al. 2021 [82]	Prospective, Phase II	62	63 (29–83)	SBRT arm: Lung: 17 Liver: 10 Lymph node: 9 Bone: 2 Other: 9		SBRT arm: 1-y LC: 54.4%	
Franzese et al. 2022 [83]	Retrospective	64	56.6 (25–82)	SBRT group Lung: 53.4 Bone: 13.3 Brain: 33.3	29.2 (2.3–117.1)	SBRT group 1-y LC: 57.5%	
Singh et al. 2022 [84]	Retrospective	81	68 (18–101)	Lung: 55.2 Lymph node: 15.4 Spine: 12.8 Liver: 3.8 Non-spine bone: 2.5		1-y LC: 93.3% 2-y LC: 93.3% 3-y LC: 77.4% 1-y OS: 66.4% 2-y: 43.1%	No Grade 3 or more

SBRT: stereotactic body radiotherapy; mo: months; DFS: disease-free survival.
